# Disabled, invisible and dismissed—The lived experience of fatigue in people with myeloproliferative neoplasms

**DOI:** 10.1002/cnr2.1655

**Published:** 2022-06-15

**Authors:** Ashleigh Bradford, Ken Young, Ashley Whitechurch, Kate Burbury, Elizabeth Jane M. Pearson

**Affiliations:** ^1^ Department of Health Services and Implementation Research | Department of Nursing | Department of Clinical Hematology Peter MacCallum Cancer Centre Melbourne Victoria Australia; ^2^ Department of Clinical Pathology | The Sir Peter MacCallum Department of Oncology The University of Melbourne Melbourne Victoria Australia; ^3^ MPN Alliance Australia Sandringham Victoria Australia

**Keywords:** experience, fatigue, MPN, myeloproliferative, qualitative

## Abstract

**Background:**

Myeloproliferative neoplasms (MPNs) are rare haematological cancers. Several studies report the most common MPN symptom leading to reduced quality of life is fatigue. Yet, how fatigue affects the lives of people with MPN is not well described.

**Aims:**

The purpose of this qualitative study is to better understand the lived experience of fatigue associated with MPN.

**Methods and results:**

People with MPN who had experienced fatigue were invited to complete an online survey and if eligible, then to participate in semi‐structured interviews and focus groups, exploring their experiences of fatigue. Thematic analysis of interview transcripts by two researchers produced themes describing the lived experience of fatigue. Twenty‐three people with MPN participated in seven interviews and four focus groups. Qualitative data revealed how fatigue significantly affected participants' experiences of functional, social, family and emotional wellbeing. Participants reported that fatigue was infrequently acknowledged or addressed by health professionals, and a lack of information or support to manage their fatigue. Four themes including 12 sub‐themes describe the experience of fatigue in MPN: (1) the distress of the MPN diagnosis, (2) sensations of fatigue, (3) daily life and emotional burden with fatigue and (4) how people managed their fatigue with limited guidance.

**Conclusion:**

Fatigue in MPN is common, debilitating and distressing. It affects all aspects of health, wellbeing and life. Health professionals could affect patients' lives substantially by acknowledging and understanding fatigue in MPN, including contributing factors and potential opportunities for management. More systematic data describing the causes and management of MPN fatigue is needed.

## 1 INTRODUCTION

Myeloproliferative neoplasms (MPNs) are a group of rare haematological cancers first classified in 1951. The estimated global incidence of MPN is 0.3–0.6 per 100 000/year, diagnosed at a mean age of 68 years.^1^ Due to their severe symptom burden, MPNs are accompanied by significant rates of morbidity and mortality.^2^ The most common MPN subtypes myelofibrosis (MF), essential thrombocythemia (ET) and polycythemia vera (PV) are referred to as the ‘classical’ MPNs.^1^ Symptoms and presentations of MPNs vary within and between subtypes, often entailing a broad array of symptoms including fatigue, itching, night sweats, bone pain and abdominal discomfort.^3^ These negatively affect the lives of patients and their families. The clinical course of MPNs is unpredictable. All MPNs can progress to acute myeloid leukaemia, which is often the final stage in the patient's disease process.^4^ As each subtype of MPN can evolve into another subtype, diagnosis, risk assessment and therapeutic choices are often challenging.^5^, ^6^

Fatigue affects up to 95% of people with MPN and is the most commonly reported symptom.^2^, ^7^, ^8^, ^9^, ^10^, ^11^ The term ‘MPN fatigue’ refers to fatigue as a symptom of an MPN diagnosis and it is often considered part of the broad category of cancer‐related fatigue.^2^ The impact of MPN fatigue is variable across patients. This may be due to the multiple potential contributing factors in a person's lived experience, which add complexity to mitigating fatigue.^2^, ^9^, ^11^ Understanding these biological and non‐biological factors could inform fatigue management strategies.

Current understanding of MPN fatigue is dominated by quantitative survey results. These have identified domains of life impacted by fatigue, including social life, relationships, work, mood, sleep patterns, life‐plans and overall quality of life (QoL).^2^, ^9^ However, academic literature lacks deeper insight into precisely how fatigue affects the everyday experiences of people with MPN, or how they live with fatigue. The purpose of this study was to gain deeper understanding of the experience of MPN fatigue, and the strategies people use to combat fatigue.

## 2 METHODS

Qualitative methodology using multiple methods explored the research question: “What is the lived experience of fatigue for people diagnosed with an MPN?” The study was grounded in phenomenology, a paradigm used in qualitative research that supports exploration of human experiences in order to learn.^12^ The research question called for a descriptive approach, whereby researcher beliefs, attitudes and expectations are set aside.^12^ Therefore, the study was not driven by pre‐determined theory, nor did it attempt to create one, consistent with descriptive phenomenology.^12^ Narrative inquiry is a methodology that is interwoven with exploring phenomena and informed the methods used to examine the experience of fatigue.^13^ Research methods included semi‐structured interviews to gather stories of people with MPNs experiencing fatigue, and thematic analysis to describe the essence and breadth of that phenomenon.^14^

To reach people across Australia, recruitment for this qualitative study was linked to an online quality of life survey distributed to several Australian online MPN support groups. Co‐author KY, a consumer advocate, was integral in reviewing, distributing and promoting the survey. The online survey collected participants' demographics, quality of life,^15^ MPN symptoms,^10^ fatigue^16^ and distress levels^17^ and is not reported in this manuscript. At the end of the survey, participants whose responses met the eligibility criteria (below) could express interest in the current study, involving a virtual interview or focus group (i.e. convenience sampling). In an attempt to achieve gender balance in a second survey distribution, males were specifically invited.

Eligibility criteria to ensure participant expertise in MPN fatigue included:

- Australian residents aged 18+ years, diagnosed with an MPN for at least 6 months;
- Experience of moderate–severe fatigue in the past 6 months and functional decline or impact due to fatigue, determined by responses to selected questions on the fatigue questionnaire^16^;
- To avoid adding to existing distress during virtual interviews of a sensitive nature, candidates indicating severe distress in the survey (PHQ‐4 score ≥9)^17^ were excluded.

Study eligibility was ascertained from survey data, then confirmed during a phone conversation with a student researcher (AB), who described the study and emailed the information and consent form to prospective participants. During a second phone contact verbal consent was audio‐recorded and an interview or focus group was scheduled.

Managing distress remotely with participants who were not registered patients at the cancer centre raised ethical concerns for duty of care. Medical and nursing specialists advised that supporting people who may be already distressed was more feasible with individual interviews than in a group. Therefore, participants with moderate distress (survey PHQ‐4 score of 6–8) were offered interviews. Participants with low distress (PHQ‐4 score <6) chose their preferred mode. A distress protocol was created in case a participant became distressed during interview or focus groups.^18^

Three qualitative methods were used to address duty of care and enhance inclusion of participants with low energy and potential distress.

1. Focus groups of 2–5 participants were used as a social space that enabled participants to hear and build on others' similar or different experiences, thereby deepening understanding of the phenomenon.^19^
2. Interviews allowed exploration of an individual's experiences and opinions,^20^ at the participant's convenience.
3. Interview via email correspondence enabled one participant with symptoms affecting their capacity for interview to participate. In this instance, interview questions were emailed to the participant, and follow up questions used to probe responses.

A semi‐structured interview guide based on literature and researcher clinical experience was developed. Co‐author KY's experience both as consumer and support group moderator, influenced the final questions and prompts, however the guide was not piloted due to COVID‐19 restraints. The same guide was used in all focus groups and interviews. See Online Appendix 1 for interview guide.

## 3 DATA COLLECTION AND ANALYSIS

Two researchers (EP & AB) facilitated the focus groups and interviews via secure videoconference or telephone. These were audio‐recorded. Participants were interviewed at home alone and none had a previous relationship with researchers who collected and analysed the data. During focus groups AB, a biomedical science student, was responsible for observing, ensuring technology systems were working, explaining next steps and thanking participants. EP, an occupational therapy specialist in oncology and postdoctoral researcher, led the questioning using the interview guide. Telephone interviews lasting 30–60 min were conducted by either EP or AB; focus groups took up to 90 min. In the focus groups, the facilitator (EP) invited responses in a pre‐set order to ensure all had an opportunity to contribute, and managing the group using cues and gestures was not possible in the online format.

The ordered approach to questioning for focus group participants resulted in limited opportunities to interact with each other. However, ideas expressed by a participant could be further explored by others, potentially deepening data richness.^21^ Due to limited interaction between focus group participants, data generated from interviews, focus groups and correspondence were considered sufficiently comparable to enable the different data sources to be integrated for analysis.

Inductive thematic analysis was performed in six steps as described by Braun and Clarke.^14^ (1) Audio recordings were transcribed *verbatim* by AB and checked by EP. Transcripts were emailed to participants to confirm and add comments. Participant names were then replaced with aliases. (2) Two researchers (AB and EP) independently read and manually coded the final transcripts using QSR International's NVivo™ 12.6 for Mac software (2019). Coding was based on overt rather than implied content. Positive and negative aspects of the same concept were coded together. The independent codes were compared, defined and adjusted to determine final codes. (3) The researchers independently grouped the codes to develop initial themes and subthemes. (4) Themes were compared and modified by consensus to form final themes and subthemes that were relevant to the research question. (5) Themes were defined and named. (6) Multiple extracts were included in early drafts of this report to highlight lived experiences and shortlisted by co‐authors. Final quotes included here were selected by KY (consumer advocate).

A high standard of analytic rigour was achieved by adhering to a thematic analysis checklist.^14^ Qualitative data saturation is considered to be reached when no new information addressing the research question is yielded from further interviews.^22^ Although Braun and Clarke argue the term ‘data saturation’ is incongruent with qualitative epistemology,^23^ in order to demonstrate a level of confidence in the results we conducted a retrospective analysis using a method described by Guest, Namey and Chen.^24^ This approach identified the number of unique codes in a ‘base size’ of the first four consecutive data collection events and new codes in the subsequent ‘run length’ of three events to determine the percentage of new information generated. Less than 5% new information demonstrates that data saturation was approaching at >95% confidence level.^24^ Further data events would be included if this were not achieved.

## 4 ETHICAL CONSIDERATION

This study was conducted in accordance with the Australian National Health and Medical Research Council National Statement on Ethical Conduct in Human Research (2007 and updates)^25^ and the World Medical Association Declaration of Helsinki (2013 and updates).^26^ The Peter MacCallum Cancer Centre Human Ethics Committee approved the project (HREC/63506/PMCC‐2020).

## 5 RESULTS

### 5.1 Participant characteristics

**FIGURE 1:**
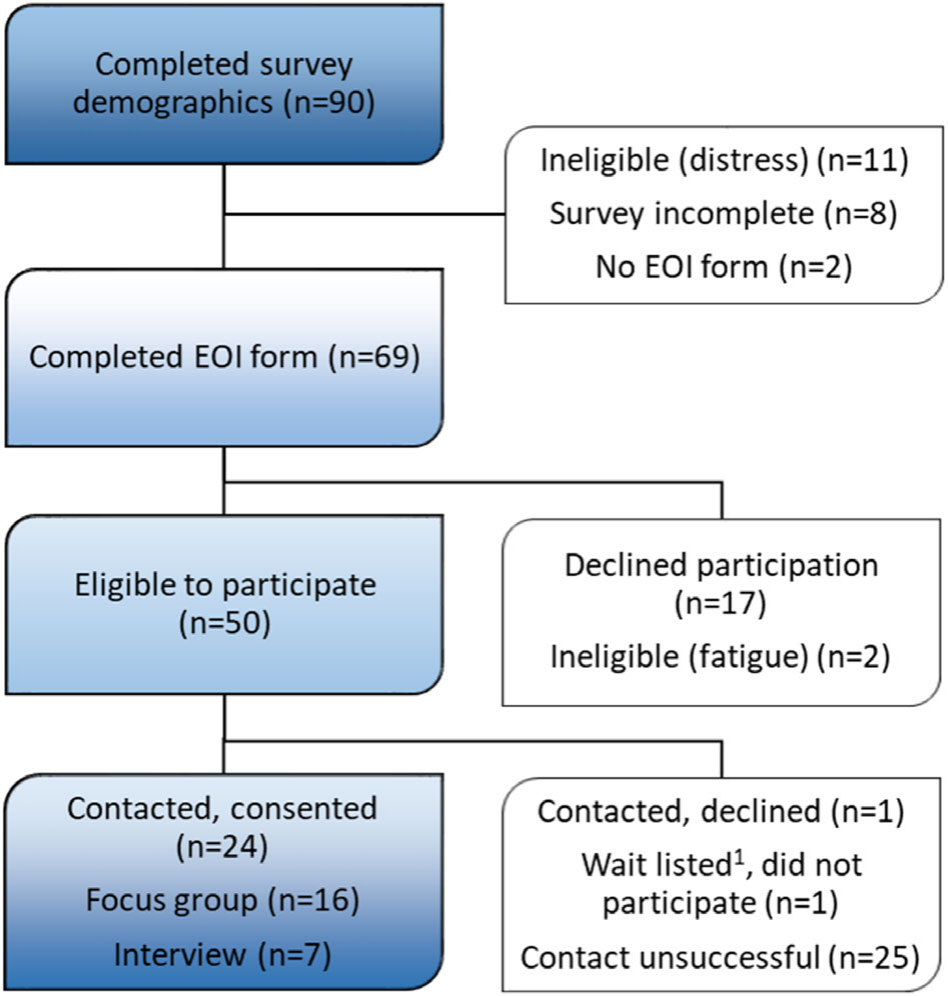
Qualitative study participant accrual. EOI, expression of interest; ^1^Wait listed, consented and placed on waiting list in case another participant withdrew.

Of 90 online survey respondents, 69 completed the expression of interest form for the current qualitative (interview) study. Eleven participants indicated severe distress and did not encounter the form. Figure 1 shows participant flow.

Table 1 summarises the age, sex, MPN type, Australian state of residence and employment status for the interview and survey participants. Approximately 75% of both survey and interview participants were female. Interview participants were marginally older, with a similar age range to survey participants. A greater proportion of the current study participants had a PV or MF diagnosis, with fewer diagnosed with ET or working than survey respondents. Participants had their MPN diagnoses between 6 months and 34 years.

**TABLE 1:** Survey and interview participant demographic information

Demographics	Survey respondents (*n* = 90)		Interview participants (*n* = 23)	
Age (years)				
Median	54.76		58.74	
Range	29.92–78.19		31.96–76.23	
Sex	*N*	%	*N*	%
Female	67	74.4	18	78.2
Male	23	25.6	5	21.7
Australian state of residence				
Victoria	29	32.2	10	43.5
New South Wales	27	30	5	21.7
Queensland	18	20	4	17.4
Western Australia	10	11.1	2	8.7
Tasmania	3	3.3	1	4.4
Australian Capital Territory	2	2.2	1	4.4
South Australia	1	1.1	0	–
MPN type (participants may have more than one MPN subtype)				
Polycythemia vera (PV)	36	40	13	56.5
Essential thrombocythemia (ET)	44	48.9	8	34.8
Myelofibrosis (MF)	14	15.6	5	21.7
Other MPN	6	6.7	1	4.4
Employment status				
Working	42	46.7	6	26.1
Not working/retired	35	38.9	13	56.5
Other, not specified	8	8.9	3	13.1
On leave	5	5.6	1	4.4

During the week preceding the survey, six (26.1%) participants in the qualitative study had experienced mild tiredness but were still able to do everything they normally do; seven (30.4%) had moderate tiredness and did less physical activity and 10 (43.5%) indicated they had severe tiredness whereby doing everyday tasks was very difficult. The mean and median scores on the FACT‐Fatigue subscale^16^ were 26.00 (SD 11.35) and 21.00 respectively (range 12.00–49.00). Scores below 34 on this instrument indicate severe clinical levels of fatigue.^27^

### 5.2 Thematic analysis of transcripts

The 23 participants included 16 who attended one of four online focus groups. Six people had an interview by telephone and one by correspondence. The 11 transcripts were independently coded by AB and EP. Data acquired in focus groups was consistent with, but generally richer than interview data. Seventy‐five initial codes were iteratively refined and defined by consensus, resulting in 61 final codes. These were organised into four main themes with 12 subthemes and 34 discrete concepts.

Data saturation analysis^24^ indicated 100% data saturation after seven transcripts (14 participants). There were 60 unique codes in the base run of four data collection events. In the next three transcripts no new codes were added (0% new information). One further code was added in the ninth transcript (20 participants). See Online Appendix 2: Chronological data events with code occurrence.

**FIGURE 2:**
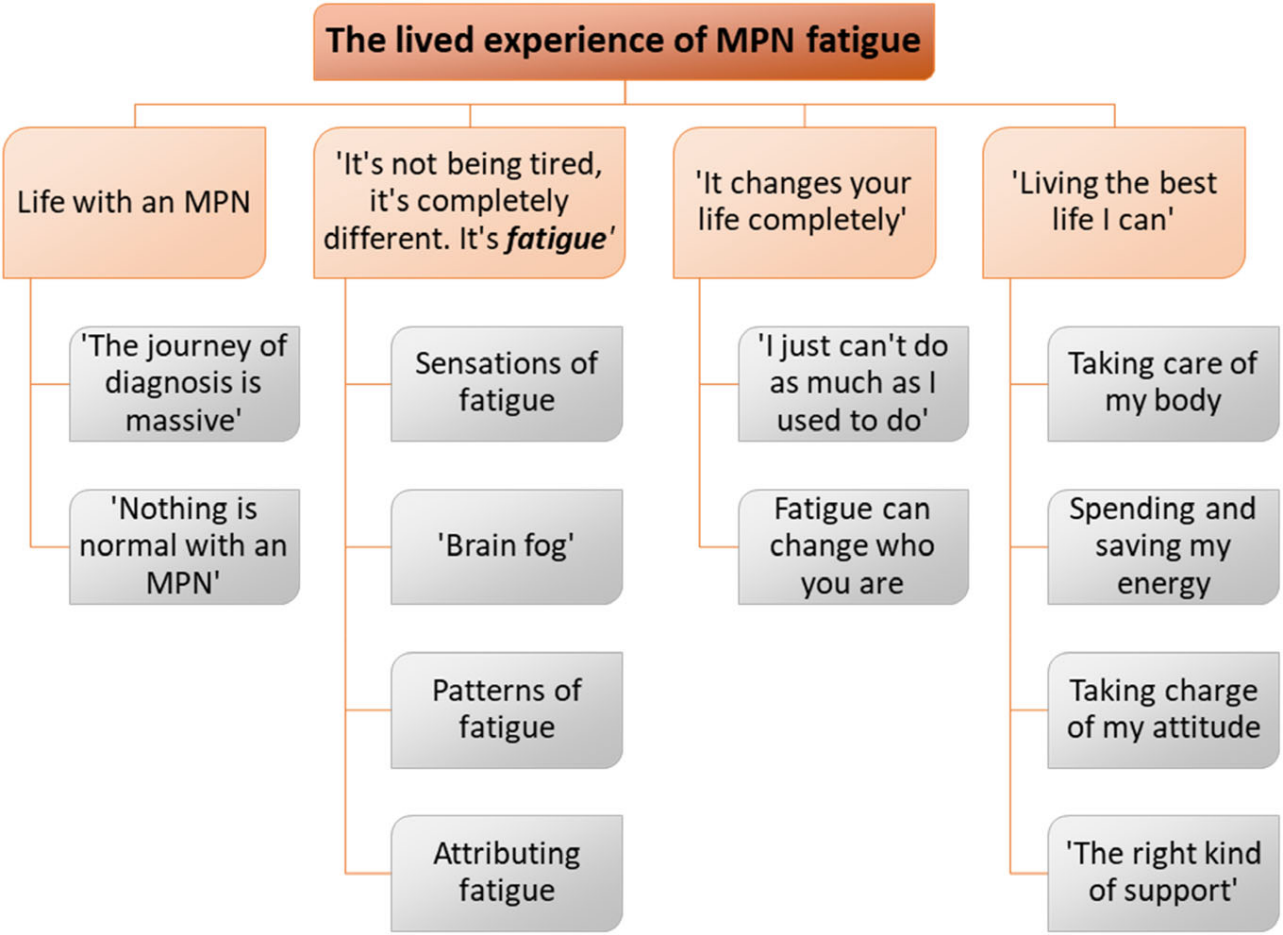
Organisation of qualitative themes and subthemes. Quotation marks denote direct quotes.

The four main themes are (1) Life with an MPN, (2) “It's not being tired, it is completely different. It is *fatigue*”, (3) “It changes your life completely” and (4) “Living the best life I can”. Quotation marks in titles indicate direct participant quotes. Subthemes provide structure and demonstrate meaning within the data. Figure 2 shows the organisation of themes and subthemes, which are described below. Detail of derivation of themes from initial codes, descriptions and frequencies for each theme, subtheme and concept are available at https://www.researchgate.net/profile/Elizabeth-Pearson-3/research.

*Theme 1. Life with an MPN* provides context for the lived experience of fatigue. Unprompted, participants explained how an MPN diagnosis profoundly affected their lives, and how they navigated their diagnosis. The theme *Life with an MPN* depicts how participants' everyday lives changed following diagnosis. This involved coming to terms with life‐long illness and a different kind of “normal”. Two clear subthemes were determined: “*The journey of diagnosis is massive*” and “*Nothing is normal with an MPN*”.

Theme 1.1. *“The journey of diagnosis is massive”* illustrates experiences of being diagnosed with an MPN either suddenly (e.g. following a routine blood test), or following a protracted period of unexplained symptoms—including fatigue

*The chronic fatigue can be crippling. It was that and the itching that led me to investigate what was going on* (Lucy, PV, 54 years)

People with months or years of symptoms often received their MPN diagnosis with relief, while for asymptomatic participants their diagnosis came as a shock.

*… Pretty shocked I suppose when you talk about what it is and you're at 45* (Nicholas, PV, 49 years)

Participants spoke of needing to understand their rare condition and seeking reassurance that they were receiving optimal treatment. They wanted to feel as well as possible and live a normal life. However, several noted disappointment in their specialists' limited professional interest in managing symptoms of their chronic illness.

*I was finding that for all the questions I had*, *the haematologist wasn't sufficiently up to speed. So*, *I've spent half my life on the internet trying to find the answers myself* (Stella, PV, 70 years)

Theme 1.2. *“Nothing is normal with an MPN”* describes a changing lifestyle due to symptoms, treatment and emotions related to living with an MPN. Participants recounted how ‘invisible’ symptoms, such as itching, pain and fatigue, were often not acknowledged by others, and difficult to explain or understand. When people commented ‘how well you look’, participants felt that others did not believe, or had forgotten they were unwell. Some participants then doubted their own symptoms or felt like imposters during hospital visits. Having to frequently explain their condition was frustrating.

*… it's lovely for people to say ‘gee you look well’*, *and it can be actually really annoying because you're thinking to yourself … I'm so tired I feel like crap and all I hear is how great I look*. (Jill, PV, 58 years)

Participants described how living with an MPN can be an emotional journey of anxiety and unease, with concerns for worsening health in the setting of an incurable condition.

*I'm quite young for someone with an MPN so I find looking forward to the future you feel a bit … sort of despairing I suppose because you know ‐ if I'm going to live my life feeling like this?*, *or it's going to get worse?* (Sarah, PV, 31 years)

Study participants made subconscious or proactive lifestyle changes to accommodate symptoms, treatments and limitations, and improve physical and mental wellbeing. Such changes could emphasise loss of health and abilities, adding to the emotional burden.

*Look*, *having an MPN means everything is adapting. You know*, *for me it's changing how I eat*. (George, MF, 69 years)

*Theme 2. “It's not being tired*, *it's completely different. It's fatigue”* portrays the qualities of MPN fatigue. Participants differentiated fatigue as more intense than regular tiredness and wanted others to understand their fatigue.

*Calling it ‘tiredness’ is hopeless. It's like people who have migraines being told they have a headache* (Lucy, PV, 54 years).

*… there's tiredness and there's fatigue. Tiredness can be fixed by going to sleep*, *fatigue can't be. You know*, *you can wake up*, *and you might feel a bit less tired*, *but you don't feel less fatigued*. (Samuel, MF, 71 years)

Qualities of MPN fatigue were characterised into four subthemes: *Sensations of fatigue*, *Patterns of fatigue*, *“Brain fog”* and *Attributing fatigue*.

Theme 2.1. *Sensations of fatigue* details bodily perceptions that infiltrated movements and actions. Fatigue was often portrayed as a whole‐body sensation, or ‘heaviness’, that left participants feeling dizzy, drained, weak, clumsy or unable to move

*I feel pretty much semi‐conscious … a sensation that I can't really move* (Tayla, ET, 58 years)

*It's a deep aching*, *overwhelming*, *profound … way of being that is just ‐ so destructive*. (Emily, MF, 44 years).

Theme 2.2. *Patterns of fatigue* describes the variable onset and severity of fatigue. Fatigue could be intense for long periods (months), or fluctuate throughout the day. Many participants reached a point when they could not continue, and they had to rest or sleep.

*Sometimes come one o'clock in the afternoon I've just got to put my feet up*, *I just can't keep going* (Victoria, PV, 63 years)

Despite resting, many participants described feeling exhausted on awakening, and it took a long time to get going.

*… you wake up in the morning and you just feel awful. Like you haven't slept at all*. (Gabriella, MF, 63 years)

Some participants felt fatigue building throughout the day, but for others fatigue was omnipresent. Many participants described a sudden awareness of fatigue, with an urgent need to sit, lie down or sleep.

*I can be feeling good and all of a sudden it will just hit me like a ton of bricks* (Ella, PV, 56 years).

*I have a lot of energy in the morning … by 3 o'clock in the afternoon I would just be so spent … and I could not do anything except just sit down and chill*, *and that just wasn't me*. (Eliza, MF, 58 years)

Theme 2.3. “*Brain fog*” refers to various cognitive effects, often experienced when fatigue was greater. Participants in every interview and focus group described difficulties with concentration, memory, word finding, feeling ‘spacey’ and ‘jet‐lagged’

*It affects me to the point where I can't concentrate. I'm constantly tired and more muddled up. You know*, *you're left confused* (Isabel, PV, 50 years)

*… you just have trouble just stringing together maybe some logical thoughts*, *certainly conversation and stuff like that*, *you can be a little bit disjointed*. (John, MF, 50yrs)

When experiencing ‘fog’, participants lacked focus for task completion, tasks took longer or they made careless errors, even as profound as leaving cash in an auto‐bank machine. They described struggling with complex work and domestic tasks, and memory lapses. Cognitive symptoms were coupled with distress, confusion and frustration, often due to not linking ‘brain fog’ with fatigue. Some participants worried they had early dementia.

*… my fatigue was bad and I was driving home not even remembering how I got home …* (Emily, MF, 44 years)

Theme 2.4. *Attributing fatigue* describes how participants rationalised their fatigue, often in a knowledge vacuum. Fatigue was attributed to age, work, exertion, blood parameters or altered body clock. Other symptoms such as pain and anaemia, MPN treatments, dietary habits or ambient temperature were noted to affect fatigue severity. Some participants perceived that hydroxyurea (treatment medication) increased fatigue and brain fog; others associated greater fatigue with interferons (immunotherapy treatment). Venesections (bloodletting) could help or worsen fatigue.

*I've had … 4 venesections at fortnightly intervals. So from feeling pretty well … hardly any fatigue*, *I'm now back in my zombified fatigue state* (June, PV, 68 years)

*… after I have a bloodletting … I always feel a lot better but I would definitely say of course I'm not back*, *I'll never be … back to the person … the levels of energy that I had*. (Jill, PV, 58 years)

*Theme 3. “It changes your life completely”* encapsulates fatigue's profound impact on participants' lives and identity, with two subthemes: *“I just can't do as much as I used to do”* and *Fatigue can change who you are*.

Theme 3.1. *“I just can't do as much as I used to do”* explains how fatigue restricted everyday tasks from self‐care to work and social life. Although often able to physically carry out many activities, participants reported reduced stamina and interest. With severe fatigue, activity tolerance was low and participants would need to rest after a short time doing a light activity.

*I'd start dead‐heading some roses*, *which is not gardening in the truest sense of the word*, *so I'd start feeling weak at the knees and I'd have to go and sit down* (June, PV, 68 years).

Some participants could only do one thing each day in addition to their self‐care, such as an appointment or household task. Getting themselves out of the house could be a huge effort.

*Just to get ready and get to a medical appointment is tiring*, *and on one occasion I fell asleep in the waiting room. So the fatigue has affected most avenues of my life*. (Sandra, PV, 75 years)

Almost all participants described limited capacity for social activities, particularly in the evening. Those able to work observed that tasks requiring greater focus, complex processes, or maintaining stamina were often difficult.

Theme 3.2. *Fatigue can change who you are* illustrates how fatigue affected emotions, behaviours, relationships and aspirations. Participants expressed sadness, grief and frustration due to losses, changing abilities and inability to live as fully as previously. Many noticed how these emotions influenced their behaviour, describing being more tense, irritable or impatient. Frustration could lead to tears, and exhaustion brought on apathy.

*I do get teary … very easily … about a week ago nothing would go right*, *I'd knock over a drink of water*, *I'd drop something and yeah I'd just end up in tears*. (Victoria, PV, 63 years)

Participants adapted their behaviour, priorities and goals to their fatigue. Many approached social activities more reluctantly, often with reduced enjoyment. Extra rest was needed beforehand and recovery sometimes lasted days. Maintaining friendships could be difficult, and repeatedly explaining their situation was tedious.

*… you feel like you can't just can't keep saying all the time*, *oh you know it's ‘I'm ok*, *I'm doing alright’ … you get sick of listening to yourself as well*. (Jill, PV, 58 years)

Some participants prioritised work over other life domains for financial reasons, or sense of contribution. Working and commuting typically exhausted their energy. Increased time resting reduced participants' ability to contribute to household tasks and intimate relations. This caused some relationships to break down, but a shared understanding of MPN and fatigue was perceived to strengthen close relationships.

*… getting home and either going straight to bed or … bombing out on the couch*, *and for me too that's where things started to disintegrate with my relationship because there's the financial implications of not being able to work*. (Emily, MF, 44 years)

*When I was diagnosed …we were engaged … he actually left me because he couldn't deal with me being unwell. Um*, *but I now have a new partner who's actually really supportive*, *and he's really good*. (Sarah, PV, 31 years)

Many participants changed their family goals and plans for travel, retirement, sport and career, because of fatigue. Some expressed gratitude for what they could still achieve.

*We make choices and still can do a lot of stuff*, *but no absolutely*, *it changes your life completely*. (Stella, PV, 70 years)

The theme *“It changes your life completely”* captures the far‐reaching impacts of fatigue on what participants could do, be and become.

*Theme 4. “Living the best life I can”* describes participants' struggle to manage their lives with fatigue. Many stressed that any approach should suit the individual. Self‐management efforts are summarised in four subthemes entitled *Taking care of my body*, *Spending and saving my energy*, *Taking charge of my attitude* and *“The right kind of support”*.

Theme 4.1. *Taking care of my body* includes measures to improve overall health and fitness. Minimising exacerbating factors such as pain and distress; and doing physical activity such as running, cycling or walking were said to help physical and mental fatigue. However, exercise could be difficult when fatigued. Several participants made dietary changes to boost their energy—eating healthier foods, increasing water intake, avoiding alcohol or following a ‘training diet’.

*I've been … reducing sugar and carbs … a fairly significant reduction and I have really noticed that my condition has improved and that my fatigue is not as bad… there's very little written about it. If you go looking online it's just not there*. (Penny, PV, 51 years)

Theme 4.2. *Spending and saving my energy* conveys how participants managed their energy to live with fatigue. Many prioritised extra sleep or rest to prepare for, and recover from activity. Resting was not always convenient or possible, but ‘pushing through’ fatigue was generally disastrous, with only one participant endorsing this approach. Planning activities to suit energy peaks and pacing activities throughout the week was helpful for many participants.

*I try to plan my days*, *by balancing different chores across several days instead of trying to do “harder work” like changing sheets on bed & washing*, *vacuuming all on one day*. (Sandra, PV, 75 years)

Some participants simplified their lives to require less energy. Examples include moving to a smaller or single level home, having a smaller garden, reducing workloads or working from home.

Theme 4.3. *Taking charge of my attitude* involved participants adapting their outlook, to reduce the emotional impact of fatigue. Some participants practised kindness to themselves, consciously letting go of negative emotions about their reduced capacity, and embracing ‘feel‐good’ activities, yoga or hobbies. Those learned to respect their capabilities and life with fatigue, and to judge themselves less harshly.

*You know there's a lot of personal work I've had to put in*, *in terms of letting go of self‐judgement … ‘I'm not good enough’*, *‘I'm not getting things done’ and all that sort of stuff. You know I'm just like ‘oh*, *ok*, *this is where I'm at today’* (Tayla, ET, 58 years)

A few participants reported psychological therapies or mind–body practices helped their mind‐set.

*I've done a little bit of meditation … it just helps you reflect*, *on your response to things and you can put things in perspective and you can decide to take control* (Nicholas, PV, 49 years)

Theme 4.4. *“The right kind of support”* describes other peoples' responses to participants' fatigue. All agreed that help was limited by others' poor understanding of the meaning and impact of fatigue. Unsatisfying responses of friends, family and healthcare professionals often led participants to online support. Many participants reported distress when health professionals handled their fatigue with disdain or disinterest.

*So they'll ask me about symptoms and I'll underreport it or not report it at all because they'll tell you that*, *‘oh well you shouldn't be having it because you're on medication and your cell counts are ok’*, *so it just got to the point where you stop reporting it because it's being dismissed*. (Sarah, PV, 31 years)

Finally, participants hoped this study would help others, particularly health professionals to better understand the experience of MPN fatigue.

*To consider patient experience … because it puts a human face and emotion … is really important to us and our families and carers and all sorts of people because … sometimes the frustration*, *or the sense of hopelessness or whatever*, *like asking professionals and not getting information or empathy*. (Lucy, PV, 54 years)

## 6 DISCUSSION

This study deepens understanding of what it is like to live with MPN fatigue. Four qualitative themes depict the struggles of an MPN diagnosis and fatigue's complex qualities and life‐changing impacts. Fatigue coupled with a diagnosis of MPN involved an emotional journey including struggle, change and acceptance. Poor stamina, yet not appearing ‘sick’ affected participants' identity, social lives and relationships. Lack of knowledge and professional guidance escalated their distress. This was also reported in an Italian content analysis of written narrative responses of people with MF.^28^

The authors believe this is the first qualitative research to be published exploring the lived experience of fatigue in MPN. While some studies have used qualitative methods to explore aspects of life with MPN such as the impact of MF on the lives of patients and carers,^28^, ^29^ these results were predominantly reported as numerical data, giving limited insight into lived experiences. Three participant quotes about the fatigue experience in MF that are congruent with our findings in Theme 2 were reported by Mesa, Su et al.^29^ Additionally, coping strategies for living with MF, aligning with subthemes 4.3. *Taking charge of my* attitude and 4.4. *“The right kind of support”* were reported by Palandri, Benevolo et al.^28^ Our study broadens this understanding.

Qualitative studies in other conditions such as chronic fatigue syndrome and cancer have likewise reported changed self‐identity, ‘invisibility’ and anxiety due to fatigue.^30^, ^31^ Qualities of cancer fatigue, including feeling different to ‘normal tiredness’, involving the whole body, cognitive effects and patterns of fatigue^32^ are strikingly similar to our findings with MPN. A meta‐synthesis of fatigue studies in rheumatoid arthritis highlighted the difficulties of communicating one's fatigue to family and friends, with similar physical, emotional, cognitive and social impacts to MPN fatigue to the current study.^33^ It seems possible that the experience of fatigue is a phenomenon that is independent of its cause. Self‐care strategies used by participants reported in subthemes 4.1. *Taking care of my body*, 4.2. *Spending and saving my energy and* 4.3. *Taking charge of my attitude* suggest the impact of fatigue can be modified by biopsychosocial factors. Our qualitative findings align with previously reported associations between fatigue severity in MPN and symptom burden, and inversely with self‐reported exercise.^2^ Together, these point to use of established guidelines for cancer fatigue for fatigue in MPN including symptom management, physical activity and cognitive approaches.^34^

Recruitment for interview‐based research in rare conditions is usually limited to a single institution or area. During the COVID‐19 pandemic online recruitment and interview methods were applied. The virtual methods enabled participants across Australia with MPN and low energy to share their experiences from home. Most would not have been able to attend in‐person interviews. This diversity strengthened the study. The rigour of qualitative method, including independent coding by two researchers and using all data to develop the themes, is a further study strength.

The data saturation analysis supports a high level of confidence in sample size adequacy, with later transcripts adding depth to themes and subthemes. Survey demographics allow appraisal of generalisability of the qualitative results. Study participants were members of online MPN support groups, and had low to moderate distress scores. People indicating severe distress or outside the virtual MPN community may have provided different perspectives. We note the lower proportion of males in both the survey and interview groups (25%) than in other MPN studies (40%).^2^, ^35^ Recruitment via online support groups may have contributed to this limitation, as women use online support groups more readily.^36^ Further, virtual focus groups and interviews may have been unpalatable to some people. This may have contributed to the lower mean age of our participants (60 years) compared with the general population of MPN patients (68 years). These sampling limitations suggest caution in generalising to the broader MPN population. We suggest including participants with different demographic characteristics in future efforts in this space, to complement our results. Lastly, it is possible that beliefs of the lead researchers EP (occupational therapist) and AB (science student) influenced the interview style, analysis and/or conclusions. We have presented our findings to a subgroup of participants who endorsed the themes and conclusions. Overall, we believe these limitations do not detract from the findings of this study.

Fatigue in MPN is an all‐encompassing, physical and emotional phenomenon that limits a person's capacity and potential. People diagnosed with MPN have struggled to deal with their fatigue with limited knowledge and professional support. It is imperative that clinicians acknowledge and address this most prevalent and debilitating MPN symptom, to achieve the best quality of life for their patients. In the absence of MPN‐specific fatigue guidelines, interventions for cancer fatigue are likely to be effective. These include reducing symptom burden, exercise and cognitive behaviour therapy,^34^ and can be delivered in primary care. Future studies investigating fatigue interventions in MPN populations and male experiences of MPN fatigue are needed to facilitate holistic treatment of people diagnosed with MPNs.

## AUTHOR CONTRIBUTIONS

**Ashleigh Bradford:** Formal analysis (equal); investigation (equal); methodology (supporting); visualization (lead); writing – original draft (lead); writing – review and editing (lead). **Ken Young:** Investigation (supporting); methodology (supporting); resources (lead); writing – review and editing (supporting). **Ashley Whitechurch:** Methodology (supporting); writing – review and editing (supporting). **Kate Burbury:** Conceptualization (supporting); methodology (supporting); writing – review and editing (supporting). **Elizabeth Jane M. Pearson:** Conceptualization (lead); formal analysis (equal); investigation (equal); methodology (lead); project administration (lead); supervision (lead); writing – original draft (equal); writing – review and editing (lead).

## CONFLICT OF INTEREST

The authors have stated explicitly that there are no conflicts of interest in connection with this article.

## ETHICS STATEMENT

This study was conducted in accordance with the Australian National Health and Medical Research Council National Statement on Ethical Conduct in Human Research (2007 and updates) and the World Medical Association Declaration of Helsinki (2013 and updates). The Peter MacCallum Cancer Centre Human Ethics Committee approved the project (HREC/63506/PMCC‐2020).

## Supporting information


**Appendix 1** Semi‐structured interview guide

**Appendix 2** Chronological data events with code occurrence

## Data Availability

Ms Bradford's (Hons) thesis is available at https://doi.org/10.13140/RG.2.2.19781.37604. Non‐identifiable interview transcripts may be made available upon reasonable request to Dr. Pearson.
