# Supplementary material for: Disabled, invisible and dismissed—The lived experience of fatigue in people with myeloproliferative neoplasms
Source: Cancer Rep (Hoboken). 2022 Jun 15;6(1):e1655. doi: 10.1002/cnr2.1655 (PMC9875604; doi:10.1002/cnr2.1655)
Supplement: Supplementary file 1 — Appendix 1 Semi‐structured interview guide [file CNR2-6-e1655-s002.pdf]

**Overview of focus group / interview topics**

The purpose of the focus groups / interviews is to gain an understanding of the impact of fatigue in people with MPN and how they have coped with it.

Focus groups and interviews and will follow the same questions, simplified for the interviews. Interviews will be offered for consumers who require an interpreter, or prefer not to participate in the online group. Interviews will be up to 60 minutes while focus group may be up to 90 minutes long.

Materials needed: Audio-recorder

**Structure of the focus group**

1. *Introduce facilitators and answer any questions about the PIC and sign consent if needed (5 mins)*
2. *Introduce purpose and rules of focus group (5 mins)*
3. *Introduce participants with name, where they are from, MPN diagnosis (5 mins)*
4. *Questions and discussion (60-70 mins)*
5. *Conclusion (5 mins)*

**Questions and key prompts**

| **Question** | **Key Prompts** |
|---|---|
| <u>The experience of fatigue</u><br>1. How would you describe your own fatigue? | What does it feel like?; How does it affect your mental and physical functioning?; How does your tiredness vary over time; When did you first notice your fatigue? |
| <u>The impact of fatigue</u><br>2. How has fatigue affected your daily life? | What has changed about the way you do things? E.g. self-care, household tasks |
| 3. Has fatigue affected your relationships? In what way? | How has fatigue affected the social events you can attend? |
| 4. Has fatigue affected your mood or outlook on life? In what way? | How have your emotions about fatigue changed over time? |
| 5. How has fatigue affected your future planning and goals? | Examples: travel, career, family plans, etc. |
| <u>Advice and self-help for fatigue</u><br>6. What advice have you had about managing fatigue? | Was the advice helpful?; Have you felt comfortable/able to discuss fatigue with your specialist or GP? |
| 7. How have you tried to help your fatigue? What worked for you? | What strategies would you recommend to others in a similar situation?; What do you wish you had known about the symptom of fatigue? |

**Detailed script**

1. **Introduce facilitators and answer any questions about the PIC and sign consent if needed (5 mins)**

"Hi I am <name> and this is <name> from Allied Health and Cancer Experiences Research. My role in this project is <Principal Investigator /assistant/student researcher> for this project. The aim of this project is to provide understand the lived experience of fatigue in MPN, to hopefully inform future resources and support services.

Thank you for agreeing to participate in this focus group. Before commencing today I need to check that you all have read and understood the Participant Information Sheet. Are there any questions about being involved in this study?"

Just a reminder we are audio recording this <session>, checking you are all happy with that.

The focus group today will last for up to 90 minutes. The questions we will ask you are about your overall experience of fatigue, the impact that fatigue has had on different aspects of your life and how you have managed your fatigue.'

I want to remind you that the information discussed today will be recorded then transcribed into a written document and used as research data. Your treating team will not be informed about what you say individually however group results will be reported. If you discuss this more widely with health professionals and other patients, please respect other focus group participants by ensuring names or disciplines are not used. Remember that when discussing this session, please be aware of other participant's rights to confidentially. This means not using any identifying features such as their name or where they live. Whilst we are recording this session for the purposes of this study, it is important that you do not record it."

Unless there are any further questions about this process, I will begin recording.

2. ***Introduce purpose / topic of focus group (5 mins)***

*Preamble.* "When talking about MPNs, a high symptom burden often springs to mind. Much research has centred around the different types of symptoms, where there lies a general consensus amongst the research that fatigue is one of the most common and severe MPN symptoms, regardless of subtype. However, not much research has focused on the lived experience of fatigue in people with MPNs".

"The main purpose of today's focus group is to discuss the ways in which fatigue impacts your life, how you experience it every day and what you have done to cope with fatigue.

### 3. Introduction to participants (5 minutes)

Before we start, it would be nice to go around and introduce ourselves to everyone by stating our first names, where we are from and your MPN diagnosis.

### *4. Questions and discussion (60-70 minutes)*

"If you have no further questions about how this focus group will proceed, lets start with some questions about your individual experiences of fatigue. Firstly, **how would you describe your own fatigue?**"

> Prompts: "What does it feel like?", "How does it affect your mental and physical functioning?", "How does your tiredness vary over time? – of the day, of the week?", "When did you first notice your fatigue?"

"Thank you for those descriptions. Do you have anything else to add?" After no more additions, move onto next question.

"Our next questions are about how your fatigue has impacted on different aspects of your lives. **How has fatigue affected your daily life?**"

> Prompts: "What has changed about the way you do things? E.g. self-care, household tasks, shopping, work/jobs and transport"

"Thank you. Is there anything else to add about fatigue and daily life?" After no more additions, move onto next question.

"Now, **has fatigue has affected your relationships and if so, in what way?** Some examples of the relationships to which we are referring are between friends, family, work colleagues, or with your partner, etc."

> Prompts: "How has fatigue affected the type of social events you can attend?"

"Thanks. Is there anything else to add about the effect of fatigue on your relationships?" After no more additions, move onto next question.

"Next, we are interested in **has your fatigue has affected your mood or outlook on life and in what way?**

> Prompts: "Tell me more about that (emotion)", "How have your emotions about fatigue changed over time?"; Examples of moods or emotions are anxiety, depression, frustration, hopelessness"

Before we move onto the next question, is there anything else to add about fatigue and emotions?"
"Now we want to ask about **how your fatigue has affected your future goals and planning?"**

> Prompts: Examples include travel, career, family plans, etc.

"Is there anything else to add?"

"The next two questions are related to the treatment of fatigue. The first question is **what advice have you had about managing fatigue?"**

Prompts: "Was the advice helpful?", "Have you felt comfortable/able to discuss fatigue with your specialist or GP?"

"Thank you. Can you think of any other advice you have received?"

"The last question is **what things have your tried to help you fatigue and what has worked, or not worked, for you?**"

Prompts: "What strategies would you recommend to others in a similar situation?", "What do you wish you had known about the symptom of fatigue?"

### *5. Conclusion (5 mins)*

"Our time is up. We will send you a written script of the focus group for you to check over. If you think of important things that we have not discussed, or disagree strongly, please let us know in your reply."

"You should talk about your fatigue experience outside the focus group, to family and health professionals. If you talk about a participant's opinions shared in this focus group, I'd like to remind you not to use any names to respect confidentiality. We will do the same in our reports".

"Thank you all so much for your valuable time today. I do hope you have found the experience worthwhile. After all the focus group and interviews are analysed, you will receive a report about this study. You are welcome contact [EP] or [AB] by email or phone to discuss this further."
